# Overpriced? Are Hospital Prices Associated with the Quality of Care?

**DOI:** 10.3390/healthcare8020135

**Published:** 2020-05-17

**Authors:** Brad Beauvais, Glen Gilson, Steve Schwab, Brittany Jaccaud, Taylor Pearce, Thomas Holmes

**Affiliations:** 1 School of Health Administration, Texas State University, Encino Hall, Room 250A, 601 University Drive, San Marcos, TX 78666, USA; 2 U.S. Army-Baylor Graduate Program in Health and Business Administration, U.S. Army Medical Center of Excellence, Fort Sam Houston, TX 78234, USA; glen.n.gilson.mil@mail.mil (G.G.); stephen.d.schwab.mil@mail.mil (S.S.); brittanyjaccaud@gmail.com (B.J.); tpearce95@yahoo.com (T.P.); thomas.j.holmes30.mil@mail.mil (T.H.)

**Keywords:** hospitals, pricing, quality, value-based purchasing

## Abstract

In most consumer markets, higher prices generally imply increased quality. For example, in the automobile, restaurant, hospitality, and airline industries, higher pricing generally conveys a signal of complexity and superiority of a service or product. However, in the healthcare industry, there is room to challenge the price-quality connection as both health prices and health quality can be difficult to interpret. In the best of circumstances, health care costs, prices, and quality can often be difficult to isolate and measure. Recent efforts by the Trump Administration and the Center for Medicare and Medicaid Services (CMS) have required the pricing of hospital services to be more transparent. Specifically, hospital chargemaster (retail) prices must now be available to the public. However, many continue to question if the pricing of health care services reflects the quality of service delivery. This research focuses on investigating the prices hospitals charge for their services in relation to the costs incurred and the association with the quality of care provided. By analyzing data from a nationwide sample of U.S. hospitals, this study considers the relationship between hospital pricing (as measured by the charge-to-cost ratio) and hospital quality performance as measured by the Value Based Purchasing Total Performance Score (TPS) and its associated sub-domains. Results of the study indicate that hospital prices, as measured by our primary independent variable of interest, the charge-to-cost ratio, are significantly and negatively associated with Total Performance Score, Patient Experience, and the Efficiency and Cost Reduction domains. A marginal statistically significant positive association is shown in the Clinical Care domain. The findings indicate that unlike most other industries, in medicine, higher pricing compared to cost does not necessarily associate with higher quality and, in fact, might indicate the opposite. The results of this study suggest that purchasers of healthcare, at all levels, have justification in challenging the pricing of healthcare services considering the quality scores available in the public domain.

## 1. Introduction

In most markets, higher prices generally imply increased product or service complexity as well as increased quality. For example, when purchasing a car, customers can expect to pay more for a luxury automobile, and in return, they can reasonably presume that the car they purchase is faster, more comfortable, or more reliable. However, this concept may not directly relate to conditions in the healthcare industry—particularly hospitals. In this situation, the patients may use price as an indicator of quality but may be unable to determine the actual quality of services provided for themselves [1]. Patients’ efforts to evaluate the quality of healthcare services are further confounded by the relative opacity of costs, prices, and quality within the care continuum. Each of these factors can often be difficult to isolate and measure. The concept of paying for quality is made even more complex in the healthcare industry because the patient rarely pays for the care directly and is indemnified from financial risk through third-party insurance companies. 

In the healthcare industry, the pricing of services performed in a hospital is established annually in the facility’s chargemaster. The chargemaster’s purpose is to record the services provided, determine the charges for each service, and generate hospital bills. Simply put, the chargemaster is a list detailing the full “retail” rate charged by a hospital for individual procedures, services, and goods, and it serves as the point of departure for all negotiations with commercial third-party payers and even those without insurance. Thus, the hospital chargemaster rate structure is a matter of interest to more than just health care finance staff and hospital administrators. It is equally important information of interest to payers, patients, and providers. Nevertheless, until very recently, individual chargemaster prices were difficult to obtain. Government agencies and politicians recognized the need for policy to improve price transparency [2]. In October 2017, the Trump Administration, via Executive Order 13813, initiated the requirement for “… hospitals to publicly post standard charge information, including charges and information based on negotiated rates and for common or shoppable items and services, in an easy-to-understand, consumer-friendly, and machine-readable format using consensus-based data standards that will meaningfully inform patients’ decision-making and allow patients to compare prices across hospitals” [3,4]. In 2018, Centers for Medicare and Medicaid Services (CMS) finalized the rule, which contained the new price reporting requirements effective 1 January 2019 and stipulated the information must be updated at least annually. Ostensibly, the new rule seeks to advance the priority of price transparency, interoperability, and burden reduction to ensure that patients have the information needed to be active healthcare consumers. Only inpatient and long-term care facilities are mandated to post these charges, and they can choose to use whatever format the organization prefers as long as the information matches the chargemaster rates [5]. 

### 1.1. Literature Review

The study of pricing information in markets is robust and continues to develop as products, services, and modalities of delivery evolve over time. In general, in a functioning market, consumers can reasonably expect to find a strong positive relationship between product quality and prices—however, this connection is not always clear. For many products and services, individuals are unable to make clear quality comparisons among available choices in the market. Quality is rarely directly discernable in any product or service, and prior researchers have shown that consumers engage in relatively little information search, even when the financial commitment involved is substantial [6,7]. Given quality is not directly observable, consumers will often consider alternative market signals of quality, including advertising [8,9,10], brand popularity [11], and price [12,13,14,15]. 

While it is an imperfect measure of quality, price can convey both supply and demand-related quality insight. Several researchers have found that consumers believe that high prices are indicators of better quality [13,14,16,17]. However, expectations of higher quality at a higher price can only be realized if suppliers do not find it profitable to “cheat” by conveying false market signals by charging higher prices for a lower quality product or service [6]. 

The price—quality relationship is particularly poignant when considered in the context of health care. Prior research indicates individuals who pay a larger share of their health care costs are likely to equate high cost with high quality and presenting cost data alongside easy-to-interpret quality information improves the likelihood that those seeking care would choose the best options [18].

### 1.2. Hospital Prices

Medical price growth is an enduring problem in the United States health care industry and is projected to accelerate in the future as prices in the overall economy increase and medical-specific price inflation grows more rapidly [19]. As previously mentioned, in the present fee-for-service dominated reimbursement system, the hospital chargemaster is the mechanism that serves as the starting point for pricing negotiations of all goods and services offered in the hospital setting. Several factors may influence the pricing of healthcare services. For example, hospitals may attempt to offset the cost of uncompensated care by raising the price for all other payers to help maintain solvency [20]. Hospitals might also set prices to have a better negotiating position when dealing with insurance companies [21]. The logic is that the higher the discount the healthcare organization can offer, the more negotiating room there is for price concessions to grant to the payer [20]. Some hospitals also admit to having no standardized method on price setting at all. For many organizations, the price is simply based on a generic formula that is calculated using past costs [22]. Other contributing factors for pricing include the number of hospital beds [12], whether the hospital is located in a rural or urban setting [21], the profit status [23], teaching status [24], the average length of stay for patients [25], the case mix index [26], and the amount of market share a hospital maintains [25]. 

### 1.3. Hospital Quality

According to The Joint Commission, quality can be defined as “the degree to which patient care services increase the probability of desired outcomes given the current state of knowledge” [27]. However, the quality of care within the United States health system lags behind other industrialized nations—especially when it comes to access to care, primary care, affordability, and equity [28]. Further, recent literature indicates medical errors may be the third leading cause of death in the nation [29]. These outcomes occur even though the U.S. spends on average twice as much per citizen on healthcare [21]. 

As difficult as pricing is to capture in the healthcare industry, quality could be considered even more challenging to grasp, not because of a lack of literature but because of the wide variety of definitions and therefore metrics that are used to measure health care quality in the hospital setting. In the past, providers, policymakers, and patients have gathered different insights about quality gleaned from mortality rates, staff-to-patient ratios, adverse events, Healthcare Effectiveness Data and Information Set (HEDIS) measures, and Hospital Consumer Assessment of Healthcare Providers and Systems (HCAHPS) data, among others [30]. In recent years, CMS has worked to define hospital quality through development of the Value Based Purchasing (VBP) data set and used these values to make quality-focused payment adjustments for hospitals that operate under its Inpatient Prospective Payment System [31]. The CMS Total Performance Score is a composite of scores drawn from four specific quality domains: clinical care, safety, efficiency, and patient- and caregiver-centered experience. Each of these domains is measured using a variety of accepted industry-wide measures and best clinical practices [31]. Due to the widespread use of Medicare as a primary insurer in the United States, over half (54%) of all hospitals are reimbursed under this system, which means that Value Based Purchasing scores are one of the few standardized quality metrics that directly ties financial incentives to quality performance [32]. 

### 1.4. Connecting Hospital Pricing and Quality

Despite the Trump Administration’s efforts to promote transparency, the question remains whether the “list prices” reflected at the hospital level can meaningfully inform patient decision-making and the “shoppability” of items and services. Few authors have studied the concepts and relationships of hospital pricing and quality in the healthcare industry on a comprehensive basis. Those that have examined this area of interest were limited in scope, focused on the variability in hospital pricing for standard procedures, or included a limited set of quality indicators. Bai and Anderson [33] studied chargemaster pricing and organizational characteristics of fifty of the highest charging (highest priced) hospitals in the country. The author found wide variation in charge-to-cost ratios with some hospitals charging as much as twelve times their own costs. These authors evaluated the characteristics of the hospitals but did not fully address the question of how much (if any) of the pricing decision made by hospitals corresponds with product or service quality. Examples of other recent work include the 2016 study performed by Gani, Ejaz, Makary, and Pawlik who found substantial variation in hospital charges for cardiothoracic and gastrointestinal procedures and reported hospitals with an extreme markup had greater perioperative morbidity [21]. In a slightly older study from 2014, White, Reschovsky, and Bond studied 2011 facilitiy claims from US autoworkers in 110 hosptials in ten metropolitan markets. Using a derived “autoworker price index” equal to the amount paid to the hospital divided by a hypothetical paid amount calculated using DRG average prices, the authors’ results showed significant price variation even within markets. The study showed high price hospitals performed better than low price hospitals on reputation-based quality measures, but higher-priced hospitals performed worse than low-price hospitals on measures of excess readmissions and patient-safety indicators, including postsurgical deaths and complications [24]. 

Building on these prior studies, our research is intended to expand the body of work that examines the relationships between hospital pricing decisions and health care quality outcomes. Our research efforts are intended to determine if there is an association between hospital pricing and the quality of healthcare. While there is not a substantial amount of literature on this topic directly related to the healthcare industry, there is an emerging consensus that healthcare pricing and the quality of care delivered—or perceived to be delivered—are either not directly connected or may be negatively associated. White, Reschovsky, and Bond [24] generally concluded high-priced hospitals performed worse in areas of measured quality such as readmission rates, patient-safety indicators, and post-surgical complications, including deaths. Even more troubling, Gani, Ejaz, Makary, and Pawlik [21] indicate some hospitals were found to increase profit from delivering a reduced quality of care. Even though each of these studies were focused on different outcome areas than our present effort, their findings lead us to the hypothesis that the higher an organization’s charge-to-cost ratio, the lower the quality of the healthcare provided across all dimensions of quality measurement—including Value Based Purchasing Total Performance Score and the sub-domains: clinical care, safety, efficiency, and patient- and caregiver-centered experience.

## 2. Methods

### 2.1. Data and Sample

Data for this analysis was supplied by the Definitive Healthcare database, which provides access to the CMS Hospital Value Based Purchasing data in addition to the numerous individual hospital organizational characteristics. The Hospital Compare VBP data used for the current study reflected calendar year 2018. The 2016 American Hospital Association Financial Module provided hospital pricing information. As we describe in the sections below, we pursued a research methodology to evaluate the association between hospital prices and quality outcomes. Nevertheless, to rule out the potential for reverse causality, we utilized older data from the American Hospital Association dataset to ensure our two datasets did not fully overlap. This allowed for the impact of hospital charges to be fully realized within the VBP reports. The practice of replacing an independent variable with its lagged value to counteract potential endogeneity has been commonly used in prior financial and economic studies [19,20]. The two datasets were linked by matching each hospital’s Medicare Provider Number (MPN). 

### 2.2. Dependent Variables

The primary dependent variable in our study is the FY 2018 Medicare Hospital Value Based Purchasing (HVBP) Total Performance Score (TPS), which is an aggregate measure comprised of four domains of quality and patient safety performance. Each of the four domains of performance are included in our analysis as secondary dependent variables. These secondary dependent variables include (a) clinical care, (b) patient- and caregiver-centered experience of care/care coordination, (c) safety, and (d) efficiency and cost reduction. In 2018, each of the four domains accounted for 25% of a hospital’s TPS, though a 33% weight may be assigned to a domain if only three of the four domains of aggregate TPS are reported to CMS. Each of the secondary dependent variables is directly linked to the primary dependent variable and was run independently when conducting the statistical analysis. The dependent variables are the unweighted (raw) measures of hospital performance and are measured on a continuous scale, with the lowest being zero and the highest possible score for each dependent and sub-dependent variable being 100. 

### 2.3. Independent and Control Variables

Consistent with Bai and Anderson [33] and Jha, Orav, and Epstein [34], we used the charge-to-cost ratio (CCR) drawn from each hospital’s Medicare Cost Reports and extracted from the Healthcare Cost Report Information System (HCRIS) database by the American Hospital Association. As these prior authors have done, we consider the CCR as a proxy for hospital pricing decisions and included it in our analysis as our primary independent variable of interest. We perceive the chargemaster, or charge description master (CDM), is the hospital-specific compilation of retail prices for all goods and services that a hospital can bill to a payer, patient, facility, or insurance company. While imperfect, at the present time chargemaster prices are the only form of hospital pricing that is fully disclosed to the public. The charge-to-cost ratio is calculated as a hospital’s total gross charges divided by its total Medicare-allowable cost or simply the inverse of the cost-to-charge variable retrieved from the 2016 AHA DataViewer Financial Module data set. The Medicare-allowable cost is the cost determined by the CMS to be associated with care for all patients, not just Medicare patients. Conceptually, if a hospital’s chargemaster rate is high compared to the Medicare allowable cost, then the charge-to-cost ratio is higher, and hospital profitability might improve. The closer the charge-to-cost is to one, the less difference there is between the actual costs incurred and the hospital’s chargemaster price. Thus, if a hospital manages to maintain or lower costs, and hospital leadership prices services to match the lower cost structure, the charge to cost ratio will move closer to one. As a clarifying example, in the Bai and Anderson article, the authors found, on average, US hospital charges in 2012 were 3.4 times the Medicare-allowable cost. In other words, when the hospital incurs $100 of Medicare-allowable costs, the hospital charges $340 [34].

Drawing from prior literature that considers hospital quality and financial performance, we included several organizational level characteristics to control for confounding variance, including the number of hospital beds as a proxy for size [35], average length of stay as a proxy for patient complexity [35], case mix as a proxy for complexity [26], hospitals’ percent of payer mix from government sources as a proxy for resource availability [35], local market wage index as a proxy for staff competency [35], hospitals’ outpatient service mix as a measure of service mix and complexity [35], rural/urban geographic location [21], for-profit ownership status [23], teaching status [24], each hospital’s market concentration as measured by the Herfindahl–Hirschman Index (HHI) [36], and each hospital’s geographic location by AHA region to account for geographic variation factors [35]. 

## 3. Analyses and Results

We used multivariate regression analysis for all models in our analysis. All statistical analyses were performed using the Statistical Package for the Social Sciences (SPSS, SPSS Inc., Chicago, IL, USA), Version 25. Table 1 provides the detailed descriptive statistics for each of our study variables. The minimum and maximum scores for each measure are retained in the table to provide perspective on the current range of performance scores within the CMS HVBP program.

Five separate regressions were evaluated pertaining to each of the five FY18 dependent variables: TPS, clinical care, patient- and caregiver-centered experience of care/care coordination, safety, and efficiency and cost reduction. Each regression model included the same control variables but only a single independent variable of interest. An alpha level of 0.05 was used as the threshold for statistical significance for all five regressions. Missing data was replaced by the variable mean, and all variables included in the analysis were evaluated for collinearity. Variance inflation factors in all analyses never exceeded a threshold of 10. Table 2 reflects the results of our analyses.

## 4. Findings

### 4.1. Descriptive Statistics

Initial assessment of the descriptive statistics of our analysis provides some interesting insights. Building on the trends noted by Bai and Anderson a few short years ago (2012), the charge-to-cost ratio values in our study continue their upward trend and exceed their maximum value of 12.6. In our study, numerous hospitals charge-to-cost ratios exceed this value, with a maximum of 16.47 and twenty-three hospitals in our sample exceeding the maximum value from the prior study. This supports their indication that price escalation continues its upward trajectory with our study showing a 4.57% compound annual growth rate in the charge-to-cost maximum values since their study was published. 

We also note the relatively low Value Based Purchasing quality score performance in our sample. On a scale of 0–100, average performance scores linger in the lower to middle range of scale, including the mean values for Total Performance Score (M = 37.17, SD = 11.18) and the secondary dependent domain variables of Patient Experience of Care, 33.91 (SD = 18.44); Clinical Care, 59.32 (SD = 19.54.76); Efficiency and Cost Reduction, 18.83 (SD = 23.99); and Safety, 53.55 (SD = 18.39). From a general health care consumer perspective, this is concerning and leads to the perception that continued focus on hospital quality performance and outcomes is warranted. 

### 4.2. Regression Analysis

Our regression findings generally indicate that higher hospital prices, as measured by the charge-to-cost ratio, are associated with lower value-based purchasing performance across several quality dimensions. In our primary analysis, hospital CCR is negatively associated with HVBP Total Performance Score (β: −0.52, S.E.: 0.12, *p* < 0.001). The Adjusted R² value for the association between FY 16 CCR and FY 18 HVBP TPS was 20.9%. One practical interpretation of these results is a one unit increase in a hospitals’ CCR is associated with a decrease in HVBP TPS of over half of a point.

Our analysis fully supports two of the secondary hypotheses suggesting the hospital CCR is negatively associated with HVBP sub-domain performance—specifically with respect to patient experience (β: −1.13, S.E.: 0.19, *p* < 0.001, Adj R² = 25.2%) and efficiency (β: −1.57, S.E.: 0.23, *p* < 0.001, Adj R² = 30.9%). Our hypothesis is marginally contradicted with respect to clinical care (β: 0.4, S.E.: 0.24, *p* < 0.096, Adj R² = 16.1%) and not supported with respect to the safety subdomain. These results can be interpreted to infer a one unit increase in a hospitals’ CCR is associated with a HVBP score decrease of over a point in patient experience score and nearly a point and a half point in efficiency scoring. 

## 5. Discussion

Our analysis appears to indicate there is a negative association between higher prices, as measured by the charge-to-cost ratio, and the aggregate Hospital Value Based Purchasing Total Performance Score quality measure. The simple indication that increased prices are only marginally associated with higher quality in one dimension of quality and may reflect poorer quality in several other areas should be of concern to health care stakeholders at all levels. When these findings are considered in the context of the lower ranges of the HVBP measure scores (see Table 1), even a half to full point adjustment constitutes a significant improvement or decline in performance. 

From a consumer standpoint, we know a rising number of patients are responsible for an ever-increasing share of their health care costs—particularly as high deductible health plans become more prevalent in the insurance market. However, simply creating more transparent pricing will not necessarily result in normal consumer shopping behavior for all healthcare goods and services. The nature of elastic versus inelastic demand for services can certainly dictate the desire or need to shop for services. Nevertheless, we contend the disconnects we have highlighted between price and quality do not make health care consumers’ search process any easier, and it may be a source of continued frustration and mistrust by patients and families in the United States. Devoid of accurate pricing and quality information—or the means to effectively connect the two—consumers may opt to rely on other factors to make their care decisions or unnecessarily avoid seeking care to their own long-term detriment. Ultimately, the opaque disassociation can obscure the care decision-making process for “shoppable” services as CMS has attempted to promote through their price transparency efforts. 

From a policy standpoint, in our judgement, the recent requirement imposed by the Trump Administration to require price transparency is well intentioned. However, based on our findings, the prices posted in the form of a chargemaster on hospitals’ publicly facing websites cannot be valued as a trusted source for quality information. We suggest the continued efforts to promote increasing granularity of price and quality information are warranted. The release of negotiated prices at the hospital level could serve to provide improved clarity of information needed to facilitate effective market interactions that can clarify decision-making, make pricing more equitable within local markets, and ultimately help drive down costs. Just as in almost any other industry, health care prices should reflect the quality of goods and services offered or, at a minimum, allow health care stakeholders the ability to make informed decisions.

## 6. Limitations and Suggestions for Future Research

There are several limitations to consider in this analysis. First, our study is single cross-section of data seeking to determine an association at one point in time. Future research might focus on determining if our studied relationship persists over time or if there are changing relationships as the industry matures within the value-based purchasing operational environment. 

Second, the CCR is an imperfect measure of hospital pricing. While imperfect, at the present time, the chargemaster is the only form of hospital pricing that is fully disclosed to the public. Unless (or until) CMS is successful at prompting US hospitals to disclose negotiated prices, we consider our approach to be a “next best” approach. Further, our study examines this relationship at an aggregate level. Future research might pursue refining the primary independent and dependent variables to reflect a more granular review of the charge-to-cost at the service line level as well as across additional dimensions of quality beyond the HVBP set of measures. More granular pricing data is warranted to better inform patients, payers, and providers care decision-making processes. At present, disclosed charge master prices do not convey as much practical meaning as one would like, since the prices disclosed tend not to be the actual prices paid by most consumers. However, this is an evolving issue as high deductible health plans and patient responsibility for the cost of care expands, and large segments of the US population remain uninsured. Fully transparent negotiated prices, as the Trump Administration has requested, would allow for a more precise examination of the association between hospital charges and quality outcomes and be of greater value to all health care industry stakeholders. 

Third, as Norton, Das, and Chen [37] and Friedson et al. [38] note, the HVBP Total Performance Score and its subdomain measures are imperfect measures of quality in the health care setting. In years of study, we have yet to identify a singular measure of quality that satisfies all stakeholders in the care delivery process. To address this issue, we selected TPS but also included the TPS sub-domains (e.g., patient safety, efficiency, clinical process, and patient satisfaction) as our dependent variables given their use by CMS in reimbursement adjustment. However, future researchers might consider incorporating a wider range of quality measures to determine if the same relationships we have found exist with respect to other measures of quality.

Lastly, our primary focus centers on the association between hospital pricing and Hospital Value Based Purchasing. However, several of our chosen control variables were found to have significant and noteworthy associations with the HVBP scores in our analysis. These findings are suggestive of areas for future research. First, facilities with government oversight and higher government reimbursement percentages perform poorly on most of the HVBP metrics apart from clinical care, indicating government run facilities require additional support and attention if improved patient care outcomes are desired policy outcome. Second, hospitals with increased outpatient service mix appear to perform better across most HVBP measures, again except for the clinical care domain. This suggests that while the migration to outpatient settings for care might improve patient safety, the patient experience, and efficiency, there may yet be room for improvement in clinical care outcomes. Third, findings pertaining to case mix reflect a marginally positive association with total performance score, significant positive associations with clinical care and patient experience, but strongly significant and negative associations with both the safety and efficiency domains. This suggests efforts to see higher complexity patients may improve clinical outcomes and may be a patient satisfier, but the average high case mix facility may also be compromising both safety and efficiency to do so. Fourth, the occupancy rate of a hospital is positively associated with the clinical care domain but negatively associated with patient experience and safety. Similar in nature to our findings pertaining to case mix, these results may indicate higher-volume hospitals may enjoy improved clinical outcomes but at the expense of patient experience and safety. Lastly, market concentration, as measured by the Herfindahl–Hirschman Index (HHI), appears to have a persistent negative association with most measures of HVBP with the exception of the safety and efficiency domains. The HHI increases both as the number of firms in the market decreases and as the disparity in size between those firms increases [36]. This appears to indicate competitive forces may have a persistently constructive association with improved performance in total performance score, clinical care, and the patient experience. 

## 7. Practical Implications

Healthcare policymakers and leaders can use our findings to highlight the apparent dissociation between hospital pricing and quality and continue to take proactive action to prompt the same market-based connections as found in other industries. Our analysis indicates that unlike most other products and services, in medicine, higher pricing, comparative to the cost, does not necessarily associate with higher quality. While the current Administration’s and CMS’s focus on hospital price transparency is warranted—and arguably long overdue—this disconnect infuses a persistent confounding influence in the patient and payer care selection decision-making process. Some authors have indicated high-price hospitals have built good reputations based on their tertiary care, but they do not execute routine inpatient care as successfully [24]. It is possible that, based on our findings, higher prices based on reputation alone may be unwarranted and may be an impediment to high quality care. Therefore, purchasers of healthcare may be better served to focus purchasing on verifiable evidence-based hospital quality scores rather than the perceived reputations or the assumption that higher pricing will equate to better outcomes or higher value.

In our judgement, high-priced low-quality care creates an undue economic burden on those with the least capacity to pay. Legislators and policy makers can also use these findings to help consider what regulations should be enacted to protect the uninsured from hospitals that demonstrate pricing behaviors not commensurate with the quality of care provided. This is especially poignant when economically fragile patient populations are considered. The health care industry generally does not expect insurers to pay the full cost of increases in healthcare chargemaster prices. Commercially insured patients pay negotiated rates based on a discount from chargemaster pricing. Likewise, publicly insured patients typically pay relatively close to a hospital’s actual cost [20]. However, vulnerable populations generally lack access to the price negotiation power of the insured population. The uninsured, out-of-network, and casualty and workers’ compensation insured patients do not have comparable bargaining influence and thus may be levied the full retail chargemaster price or a relatively high percentage of the full amount, unless the hospitals voluntarily offer discounts [33]. This group of individuals is as large as 27 million people—or roughly equal to ten percent of the non-elderly adult population in the United States—and needs easy access to price and quality assessment capabilities to clearly evaluate care options [39]. Without a sufficient degree of clarity, deferred and chronic care costs can be expected to be passed on to the commercially and publicly insured populations in perpetuity. Although transparent pricing and quality information will not completely solve the care seeking process for the most economically vulnerable, we suggest it is a step in the right direction as it at least eases the costs associated with the care seeking and evaluation process. 

## 8. Conclusions

Our study indicates that a disconnect between pricing and specific dimensions of quality persists in the United States hospital industry. The relatively low average performance scores reflected among HVPB participating hospitals coupled with chronic variation and inflation in hospital pricing combines to provide justification for continued scrutiny of this relationship. Our research findings support the continued efforts by the President, CMS, commercial payers, patients, and families to press for increasing price and quality transparency. Ultimately, sufficient pressure from these key stakeholder groups might restore the natural connection between pricing and quality that is enjoyed in other industries to the positive benefit of future healthcare consumers. 

## Figures and Tables

**Table 1:** Descriptive statistics of independent variables.

Descriptive Statistics				
Variable	Minimum	Maximum	Mean	Std. Dev.
FY18 Total Performance Score	6	87.33	37.17	11.18
FY 18 Patient Experience of Care Score	0	100	33.91	18.44
FY 18 Clinical Care Score	0	100	59.32	19.54
FY 18 Efficiency and Cost Reduction Score	0	100	18.83	23.99
FY 18 Safety Score	2	100	53.55	18.39
FY16 Charge-To-Cost Ratio	0	16.47	4.76	2.26
Number of Hospital Beds	13	2654	241.98	220.18
Average Length of stay	1.72	40.61	5.13	2.95
Hospital Case Mix	0.87	3.72	1.60	0.30
Govt Payer Percentage	0	1	0.71	0.11
Local Wage Index	0.69	1.80	0.99	0.20
Outpatient Service Mix	0	0.93	0.53	0.14
Rural Hospital	0	1	0.23	0.42
Government Operated	0	1	0.14	0.34
For Profit Hospital	0	1	0.16	0.37
Teaching Hospital	0	1	0.47	0.50
Sole Community Provider	0	1	0.09	0.28
System Member	0	1	0.73	0.45
Hospital Market Concentration Index	0.02	1	0.35	0.33
Hospital Occupancy Rate	0.09	1	0.57	0.17
Region 1 (CT, ME, MA, NH, RI, VT)	0	1	0.05	0.21
Region 2 (NJ, NY, PA)	0	1	0.12	0.33
Region 3 (DE, KY, MD, NC, VA, WV, DC)	0	1	0.09	0.28
Region 4 (AL, FL, GA, MS, SC, TN, PR)	0	1	0.17	0.38
Region 5 (IL, MI, IN, OH, WI)	0	1	0.17	0.38
Region 6 (IA, KS, MN, MO, NE, ND, SD)	0	1	0.08	0.27
Region 7 (AR, LA, OK, TX)	0	1	0.13	0.33
Region 8 (AZ, CO, ID, MT, NM, UT, WY)	0	1	0.07	0.25
Region 9 (AK, CA, HI, NV, OR, WA)	0	1	0.12	0.33

**Table 2:** Multivariate regression results.

Variable	Total Performance Score			Clinical Care			Patient Experience of Care			Safety			Efficiency & Cost Reduction		
	β	S.E.	Sig	β	S.E.	Sig	β	S.E.	Sig	β	S.E.	Sig	β	S.E.	Sig
	*N* = 2744, Adj R^2^ = 20.9%			*N* = 2744, Adj R^2^ = 16.1%			*N* = 2744, Adj R^2^ = 25.2%			*N* = 2744, Adj R^2^ = 24.5%			*N* = 2744, Adj R^2^ = 30.9%		
Charge-to-Cost Ratio	−0.52	0.12	***	0.40	0.24	+	−1.13	0.19	***	−0.02	0.18	-	−1.57	0.23	***
Beds	−0.01	0.00	***	0.01	0.00	***	−0.01	0.00	***	−0.01	0.00	***	−0.01	0.00	**
Rural	3.60	0.70	***	3.47	1.44	*	2.89	1.12	**	4.70	1.09	***	3.61	1.40	**
Government	−1.95	0.63	**	−2.34	1.31	+	−1.26	1.01	-	−1.16	0.98	-	−3.21	1.27	**
For Profit	−1.47	0.66	*	−1.45	1.37	-	−4.63	1.06	***	1.44	1.03	-	−0.46	1.32	-
Teaching	1.42	0.46	**	1.04	0.94	-	2.49	0.73	***	1.51	0.71	**	0.78	0.91	-
Outpatient Service Mix	16.13	2.07	***	−16.71	4.28	***	29.82	3.31	***	11.41	3.22	***	37.78	4.15	***
Average Length of Stay	0.15	0.07	*	−0.54	0.15	***	0.25	0.12	*	0.17	0.12	-	0.76	0.15	***
Case Mix Index	0.31	0.84	-	8.40	1.74	***	8.80	1.35	***	−9.97	1.31	***	−9.48	1.69	***
Govt Payer Percent	−13.07	1.92	***	−0.11	3.98	-	−25.87	3.08	***	−8.36	3.00	**	−16.38	3.86	***
Wage index	2.24	1.77	-	12.66	3.66	***	−5.04	2.83	-	−2.39	2.76	-	0.60	3.55	-
Sole Community Provider	2.97	0.78	***	−0.38	1.61	-	0.29	1.25	-	2.47	1.22	**	8.88	1.56	***
System Member	1.12	0.49	*	0.72	1.01	-	0.32	0.78	-	0.28	0.76	-	3.02	0.98	**
Hospital Market HHI	−2.65	0.82	***	−8.93	1.70	***	−4.14	1.32	**	−2.04	1.28	-	2.95	1.65	+
Occupancy Rate	−2.77	1.58	-	12.69	3.26	***	−10.55	2.53	***	−13.15	2.46	***	−1.45	3.16	-
Region 2	−1.52	1.13	-	−10.30	2.33	***	−4.57	1.80	**	0.20	1.75	-	9.57	2.26	***
Region 3	0.53	1.28	-	−10.41	2.64	***	−3.80	2.04	-	2.14	1.99	-	13.79	2.56	***
Region 4	−1.39	1.26	-	−13.80	2.60	***	0.70	2.02	-	−0.08	1.96	-	8.17	2.52	***
Region 5	0.91	1.12	-	−6.32	2.32	**	0.73	1.79	-	2.12	1.75	-	7.20	2.25	***
Region 6	2.00	1.26	-	−11.57	2.60	***	−1.80	2.01	-	−0.05	1.96	-	21.56	2.52	***
Region 7	−1.24	1.27	-	−12.02	2.63	***	2.16	2.04	-	1.33	1.98	-	3.68	2.55	-
Region 8	−0.46	1.29	-	−12.67	2.66	***	−5.87	2.06	**	−0.13	2.01	-	17.30	2.58	***
Region 9	1.38	1.18	-	−15.86	2.43	***	−4.22	1.88	*	1.59	1.83	-	25.69	2.36	***

Note: + *p* < 0.1, * *p* < 0.05; ** *p* < 0.01; *** *p* < 0.001; AHA Region 1 is Reference.
